# Gratitude Depends on the Relational Model of Communal Sharing

**DOI:** 10.1371/journal.pone.0086158

**Published:** 2014-01-22

**Authors:** Cláudia Simão, Beate Seibt

**Affiliations:** 1 Centro de Investigação e Intervenção Social (CIS-IUL), Instituto Universitário de Lisboa (ISCTE-IUL), Lisboa, Portugal; 2 Department of Psychology, University of Oslo, Oslo, Norway; University of Udine, Italy

## Abstract

We studied the relation between benefits, perception of social relationships and gratitude. Across three studies, we provide evidence that benefits increase gratitude to the extent to which one applies a mental model of a communal relationship. In Study 1, the communal sharing relational model, and no other relational models, predicted the amount of gratitude participants felt after imagining receiving a benefit from a new acquaintance. In Study 2, participants recalled a large benefit they had received. Applying a communal sharing relational model increased feelings of gratitude for the benefit. In Study 3, we manipulated whether the participant or another person received a benefit from an unknown other. Again, we found that the extent of communal sharing perceived in the relationship with the stranger predicted gratitude. An additional finding of Study 2 was that communal sharing predicted future gratitude regarding the relational partner in a longitudinal design. To conclude, applying a communal sharing model predicts gratitude regarding concrete benefits and regarding the relational partner, presumably because one perceives the communal partner as motivated to meet one's needs. Finally, in Study 3, we found in addition that being the recipient of a benefit without opportunity to repay directly increased communal sharing, and indirectly increased gratitude. These circumstances thus seem to favor the attribution of communal norms, leading to a communal sharing representation and in turn to gratitude. We discuss the importance of relational models as mental representations of relationships for feelings of gratitude.

## Introduction

Imagine you fall ill, and have to spend a week in the hospital. One of your acquaintances visits you almost every day. Even though she is not a good friend, you have fun and enjoy the afternoons you spend together. You had never done something similar for that person, and you do not know if you will ever have the chance to repay this favor. When you leave the hospital, you remember laughter, conversations, and reading stories to each other. Probably, you will feel grateful towards the person, appreciating the effort she made to help you over this difficult time.

This is because the person benefited you. She turned what could have been an awful experience into something almost enjoyable. So one could conclude that benefits evoke gratitude – the larger the benefit, the greater the gratitude. However, already Adam Smith pointed out [1] that not all benefits elicit gratitude. How can we then explain which benefits do elicit gratitude? Returning to the example from above, imagine you had suspected or learned that your acquaintance paid these visits in order to get something in return. You would probably feel less grateful, maybe even betrayed. This means that gratitude depends on the intentions of the benefactor, a point that has been raised and corroborated by many researchers [2]–[6].

However, other persons' intentions are not always explicitly communicated. Often, individuals just seem to know whether the other person just wants to be nice or whether she expects something in return. How does this work? Relational models theory (RMT) [7], [8] posits that individuals hold mental models of *relationships*, and that these models determine which intentions one assumes the other person to have.

### Social Relationships

According to RMT [7], social relationships are governed by cognitive models or relational mental structures. These models are universal representations of social relationships and serve to coordinate and organize social interactions [7], [9], [10]. In each of the relational models, benefits have different relational meanings. In the communal sharing model, relationships are categorized as strong bonds constituted through solidarity and unity, applying the principle of “all for one, and one for all.” Partners feel motivated to share resources and to attend to each other's needs. Thus, benefits are motivated by concerns for the partner's welfare. Equality matching is constituted through even distribution procedures and reciprocity norms. Whenever people take turns, flip a coin, or use other means to establish equality, they apply the relational model of equality matching. Benefits are used to establish symmetry between parties and are reciprocated in kind. The beneficiary is indebted until he or she can repay the benefit. Inability to reciprocate is one cue for the appropriateness of an asymmetrical relational model. For such asymmetrical cases, authority ranking is the model typically applied in hierarchical relations. The superior individual is entitled to respect, deference and tangible resources, in turn has to provide protection and help. The fourth relational model, market pricing, is not part of the present research. We discuss this in the general discussion [7], [9], [11]–[13].

Communal sharing is the only model where benefits are not given out of, or entail an immediate obligation. Thus, a benefit is either a response to a relational obligation, or entails a relational obligation for the other party. Rather, benefits are given out of a consideration of the other person's needs. Most theories of gratitude assume that this is a pre- condition for gratitude [1], [2], [4]–[6], [14]–[17]. If this is true, then representing an interaction as communal sharing is a pre- requisite for feeling grateful about a benefit. The same should not be true for equality matching or authority ranking, given their associated relational obligations.

Accordingly, we predict that after receiving a benefit, or imagining receiving a benefit, the amount of gratitude experienced is uniquely predicted by the communal sharing model, and not by any other model. Across three studies, we tested whether the extent of communal sharing perceived in a relationship predicts feelings of gratitude. We varied the nature of the benefit across studies: in Study 1, participants imagined receiving a specific benefit from a new friend. In Study 2, participants recalled receiving a large benefit and in Study 3 we manipulated the benefit in an online interaction with an unknown fellow student. Across all studies we measured relational models and gratitude. Specifically, we hypothesized that when different relational models are considered simultaneously, gratitude is predicted by communal sharing but not by equality matching (Studies 1–3) or by authority ranking (Study 1 and 2).

Apart from testing the main hypothesis across all three studies, we also tested in each of the studies one additional assumption to better understand the relationship between benefits, gratitude and communality. The first assumption concerns the relationship between communal sharing, gratitude, and one's own motivation to fulfill the partner's needs. When individuals represent a particular relationship as communal sharing, they will assume the other person tries to fulfill their needs, leading to gratitude, but they will also be motivated to fulfill the needs of the communal partner. This motivation is called communal strength [18]. Accordingly, we assume that communal sharing predicts both gratitude and communal strength independently (see also the general discussion).

The second assumption concerns gratitude in the absence of any current benefit. If one aspect of communal sharing is the expectation that the relational partner intends to fulfill one's needs, then this expectation might suffice to produce gratitude regarding the relational partner [19]. Thus, one is grateful for being taken care of by the relational partner. Therefore, we tested whether the extent of communal sharing perceived in a relation predicts future feelings of gratitude regarding *the* relational partner when no benefit is mentioned (Study 2). Finally, receiving a benefit in a situation where one cannot return it (a *non-contingent benefit*) should be a cue to communal sharing. Therefore, we tested whether receiving a non-contingent benefit induces communal sharing (Study 3).

## Study 1

In Study 1, we used a pre-tested favor scenario to evoke feelings of gratitude [20]. We hypothesized that gratitude would be dependent on the perceived extent of communal sharing in the relationship to the benefactor. Specifically, communal sharing should predict gratitude when controlling for equality matching and authority ranking, whereas the other two models should not. We also predicted that communal sharing would influence separately communal strength and gratitude. This is because communal sharing is a mental representation of a relationship based on communal norms. Interpersonal interactions are interpreted according to the applied relational model, and its relational schemes [21], [22]. Communal sharing and communal norms fit with the interpretation that the benefactor is motivated by concern for one's welfare, which should evoke gratitude. Communal norms are also suggested to precede communal strength, one's own motivation to meet the other person's needs [18]. Thus, both gratitude and communal strength should be predicted by communal sharing.

### Method

#### Participants

First-year university students were asked via e-mail to take part in an online study. Students (72% females) from a Portuguese university in Lisbon (ISCTE-IUL) and a German university in Dortmund, (University of Dortmund) took part in this study (56% Portuguese). Analyses are based on 145 participants (*M^age^*  = 21.19, *SD* = 5.41), given that ten participants were excluded from the analyses because they did not follow the instruction to name one first year student and named several. German participants received course credit for the study whereas Portuguese participants were all volunteers. All procedures were conducted according to the ethical guidelines and approved by the ethics board of the Scientific Commission of the hosting institution, Centro de Investigação e Intervenção Social (Cis-IUL).

#### Procedure and Measures

In the middle of the first semester of their first year, students were invited through the university's mailing list to participate in an online study about social interactions. After reading the informed consent, participants were asked to check a box in order to give their consent to proceed to the online study. Those who agreed to participate were asked to name one other first year student with whom they had become friends since the beginning of the academic year. This was done to control for the type and duration of the relationship by keeping them constant across participants. Participants were then instructed to read the following scenario and to imagine themselves living the situation with the person they had just mentioned:

It is the beginning of the semester and you are standing in line at the bookstore to buy all the books for your classes. You are waiting in line with a friend and both of you joke about how long this is taking. After a long wait you learn that the total cost for your books is 100€, which is more expensive than what you expected. You only have 75€ in cash and it is not possible to pay by card. As you are standing there and wondering what to do, your friend offers to lend you the extra 25€: “Don't worry, I've been in that situation before and it is a real bummer. I will lend you the money and whenever you can, you give it back to me. So you don't have to go back to the line again.” You take the offer and proceed with the purchase of your books [20].

After reading the scenario, participants filled out the following dependent measures (in each study, we asked a few additional questions to disguise the true purpose of the study. The complete material can be obtained from the authors):

Participants were asked to what extent they had felt *gratitude* towards the new friend in the imagined scenario, on a 7-point Likert-scale (1 =  not at all; 7 =  extremely). Additionally *relational models* were measured with a questionnaire from Haslam (1994) adapted to Portuguese and German. All the items were translated and back translated. Each relational model is assessed with six items (ranging from 1 =  not at all true in this relationship to 7 =  completely true in this relationship). An example item for communal sharing is “*‘What's mine is yours’ is true of this relationship”,* α = .83, for authority ranking “*One of you takes most of the initiative”*, α = .72 and for equality matching “*Your relationship is organized on a 50: 50 basis”,* α = .60 (see Appendix S1 for subscales). To finish the questionnaire, participants filled out the *communal strength measure*, a 10-item scale by Mills, Clark, Ford, and Johnson [23] which measured the communal strength of the participant towards the new friend (α = .83). In particular, the scale assesses the degree of motivation one feels to attend to a partner's needs.

### Results

Preliminary analyses indicated that there were no differences between the Portuguese and the German sample concerning our hypothesis tests, so we combined them (see Text S1 for additional analyses). We then tested which relational models predict gratitude. We regressed gratitude on communal sharing, authority ranking and equality matching. As hypothesized, communal sharing significantly predicted gratitude (β = .27, *p*<.01), and authority ranking (β = .14, *p* = .10) and equality matching (β<.1) did not.

We then tested the relation between communal strength and gratitude. As expected, communal strength and gratitude were positively correlated with each other (*r* = .23, *p*<.01). Therefore, we conducted two different regression analyses. First, we tested whether communal sharing would predict gratitude when controlling for communal strength. The results revealed that communal sharing significantly predicted gratitude (β = .29, *p*<.01), but communal strength did not (β<.1). Our second analysis was to test whether communal sharing or gratitude would predict communal strength. As hypothesized, communal sharing was the only predictor of communal strength (β = .61, *p*<.001), when controlling for gratitude (β<.1).

### Discussion

As expected, gratitude evoked by a favor scenario was predicted by the relational structure of communal sharing. The link between benefits and gratitude has been established in previous research findings [2], [5], [24], as well as the link between benefits and communal-oriented relationships [25]–[27]. The present findings show that the communal sharing model predicts gratitude, in comparison to other relational models. A second, independent effect we found was that communal sharing predicted communal strength.

In this study, we established the link between communal sharing and gratitude. However, this effect was based on self-reports about an imagined scenario. Even though we used a pre-tested scenario, we acknowledge that scenarios are less involving than actual events, which can bias reactions to them [20], [28]. Therefore, we conducted Study 2 to obtain clearer evidence for the role of communal sharing in predicting future feelings of gratitude, based on real-life benefit events. If our reasoning is correct, then gratitude will depend on the relational model of communal sharing, and communal sharing at one point in time should predict feelings of gratitude at a later point in time, whereas the opposite should not be true. This would be an indication that communal sharing is a predictor of gratitude [29].

Another goal of Study 2 was to test whether communal sharing influences gratitude regardless of whether a benefit is evoked or not. If the expectation that the relational partner intends to fulfill our needs indeed produces gratitude regarding the relational partner, as we argued in the introduction, then we should find that communal sharing predicts gratitude for a concrete benefit and a more generalized gratitude regarding the relational partner. Accordingly, simply recalling the communal partner should suffice to evoke gratitude. Therefore, we conducted Study 2 over three time points, assessing gratitude for a benefit in the first wave and gratitude regarding the relational partner later. To better understand and explore these relations we used a cross-lagged panel design.

## Study 2

In Study 2, participants were instructed to recall receiving a large benefit from someone they knew, and we assessed gratitude and the same three relational models as in Study 1. We predicted that communal sharing would predict feelings of gratitude for the benefit, replicating the findings of Study 1, and that the other relational models would not predict gratitude. In addition, we tested whether a stable communal sharing relationship would foster feelings of gratitude over time. After three and again after six weeks, we repeated all measures, this time asking about gratitude regarding the person they mentioned the first time, not regarding the benefit. We hypothesized that the relational models and gratitude would show a high stability, and that communal sharing would predict gratitude over time. Specifically, we predicted that communal sharing at Time 2 would predict gratitude at Time 3. Furthermore, we expected these effects to be unidirectional.

### Method

#### Participants

Students from a Portuguese university in Lisbon (ISCTE-IUL), enrolled in an introductory sociology class participated in this study in exchange for course credit, either at one, two or three time points. The cross sectional analysis is based on 71 participants (*M^age^*  = 26.34, *SD* = 9.34), given that three out of originally 74 participants named several persons and had to be excluded. For these 71 participants, the dropout rate over time was 19%, leaving 58 participants (73% females) for the longitudinal analysis, with an age range from 19 to 53 years (*M* = 26.60, *SD* = 9.42). All procedures were conducted according to the ethical guidelines and approved by the ethics board of the Scientific Commission of the hosting institution, Centro de Investigação e Intervenção Social (Cis-IUL).

#### Procedure and Measures

At **TIME 1** (**T1**), participants arrived at the lab, read and signed the informed consent, and were told that they were about to participate in a study about interpersonal relations. They completed the study individually at a computer. Participants were also told that they should complete follow-up measures online in two more sessions.

First, participants were instructed: “Please, describe a specific situation when someone you know did something really nice for you” adapted from Algoe and Haidt, [30]. Next, they wrote the name of the benefactor, and specified the type of relationship (e.g., parent, friend, spouse, etc.) Afterwards, participants answered the following dependent measures: *Gratitude* (“*How much gratitude did you feel in the mentioned situation?”* from 1 =  not at all to 5 =  extremely), and the relational models questionnaire from Study 1 with the three subscales *communal sharing* (α = .88), *authority ranking* (α = .73) and *equality matching* (α = .75).

At **TIME 2** (**T2**) and at **TIME 3** (**T3**), an e-mail with the links to the follow-up questionnaires reminded participants of the name of the benefactor and the type of relationship they had given at T1. This information was repeated in the beginning of each online questionnaire. Contrary to T1, where gratitude was assessed regarding the situation, at T2 and T3 we measured *gratitude* regarding the person: “*Considering the person mentioned before, how much gratitude do you feel?*” Apart from this, participants responded to the same scales presented at T1 regarding the same person – *communal sharing* (T2 α = .92; T3 α = .94), *authority ranking* (T2 α = .80; T3 α = .80), and *equality matching* (T2 α = .75; T3 α = .78). In the end, participants were fully debriefed.

### Results

#### Preliminary Analyses

We predicted longitudinal effects of communal sharing on gratitude based on the assumption of stability of communal sharing over time due to ongoing stable relationships. We predicted that there would be no difference in the reported levels of relational models from T2 to T3. This would support the assumption of relational stability over time, which is necessary for concluding that the predictor (communal sharing) influences the criterion (gratitude) over time. We decided not to include T1 in this analysis, given that T1 differed from later assessments in that participants recalled a benefit and reported gratitude for that benefit. Thus, we tested the effect of time (from T2 to T3) on all variables (communal sharing, authority ranking, equality matching and gratitude). As expected, time had no effect on any relational models (*F*s< |2.5|, *p* = *ns*) nor on gratitude (*F*<1, *p* = *ns*).

#### Cross-Sectional Effects

To replicate the main finding of Study 1, we tested whether gratitude was predicted by communal sharing after recalling a past beneficial event (T1). We regressed gratitude on communal sharing, equality matching and authority ranking. Similarly to Study 1, communal sharing (β = .33, *p*<.05), but not equality matching (β< |.13|, *p* = *ns*) nor authority ranking (β<.1, *p* = *ns*) predicted gratitude.

#### Cross-Lagged Regressions

Cross-lagged regressions test the effect of one set of variables on another over time. To test whether communal sharing influences how grateful individuals come to feel regarding their relational partner, we conducted one panel of regression analyses: predicting gratitude at T3 by all variables at T2 (see Table 1). Communal sharing and not the other relational models predicted gratitude over time. T2 communal sharing predicted T3 gratitude (β = .39, *p*<.05). Neither T2 authority ranking nor T2 equality matching predicted gratitude (βs< |.21|, *p* = *ns*).

**Table 1:** Cross-Lagged effects between gratitude, communal sharing, equality matching, and authority ranking from time 1 to time 2 and time 2 to time 3 in Study 2.

Effects	T2–T3
Gratitude	
Stability of gratitude	.50^***^
Communal sharing to Gratitude	.39^*^
Equality matching to Gratitude	−21
Authority ranking to Gratitude	−.06
Communal sharing (CS)	
Gratitude to CS	−.10
Stability of communal sharing	.86^***^
Equality matching to CS	−.03
Authority ranking to CS	.02
Equality matching (EM)	
Gratitude to EM	−.20^*^
Communal sharing to EM	.23†
Stability of equality matching	.72^***^
Authority ranking to EM	−.01
Authority ranking (AR)	
Gratitude to AR	−.09
Communal sharing to AR	−.04
Equality matching to AR	.06
Stability of authority ranking	.70^***^

*Note*: Standardized regression coefficients are given.

† *p*<.10; ^*^ *p*<.05; ^***^ *p*<.001.

When we tested the opposite direction for each relational model, gratitude did not predict any of the relational models over time: T2 gratitude did not predict T3 communal sharing (β< |.10|, *p* = *ns*) nor T3 authority ranking (β<.1, *p* = *ns*). However, T2 gratitude negatively predicted T3 equality matching (β = −.20, *p*<.05). Thus, the results showed a non-recursive model: communal sharing predicted gratitude across time, but the opposite did not happen.

### Discussion

Study 2 replicated the findings from Study 1 that gratitude for benefits is predicted by a communal sharing relational model. Moreover, neither authority ranking nor equality matching predicted gratitude, which suggests that the marginal effect of authority ranking from Study 1 was either due to chance or only occurs under a limited set of circumstances. Additionally, the results showed that holding a communal sharing model for a close relationship increases future grateful feelings regarding the relationship partner when no benefit is mentioned. Furthermore, the other path from gratitude to communal sharing was not significant, allowing an interpretation of the communal sharing model predicting gratitude. Equality matching and authority ranking had no longitudinal effect on gratitude, when controlling for all other variables. These data suggest that gratitude is more than a response to a benefit: they suggest that gratitude can be relational, driven by the representation of a relationship as communal sharing.

To corroborate this interpretation, we tested whether receiving a benefit from a stranger leads to gratitude regardless of relational model, or whether even in this situation, feeling grateful depends on applying the communal sharing model. Furthermore, by measuring current gratitude for a current benefit, we sought to eliminate potential bias due to imagination or memory. Therefore, we experimentally manipulated whether or not participants benefited from a kind act of an unknown fellow student. We tested again the main hypothesis that communal sharing, and not equality matching, would predict gratitude. In addition, we tested the assumption that benefiting would increase the likelihood of applying a communal sharing model. Giving a benefit to a stranger of equal status, who does not have any identifying information about the benefactor, – hereafter called non-contingent benefit – signals an intention to fulfill the other person's needs, because reciprocation is highly unlikely. Such an intention is typical for communal sharing, where relationship partners act out of concern for each other's welfare. The reason for this concern can be that a person considers the other as equivalent in some important aspect, for example, as member of the same group, team, or family. Therefore, non-contingent benefits are cues to communal sharing and should prompt a communal sharing model in the recipient [31].

## Study 3

Studies 1 and 2 showed that after receiving a benefit, feelings of gratitude were dependent on applying a communal sharing model to the relationship. However, these studies did not measure the effects of benefiting compared to not benefiting. Furthermore, participants answered regarding people they already knew. To exclude possible confounds, in this study the interaction partner was an unknown fictitious participant. We predicted that participants who benefited would construe the relationship more according to communal sharing than participants who did not benefit. Gratitude would not depend on the benefit directly, but on construing the relationship as communal sharing.

We therefore manipulated whether the participant was the target of a non-contingent benefit (self-related benefit) or whether another fictitious participant was the target of the same benefit (other-related benefit) and the reason for the benefit. We measured gratitude, communal sharing, and equality matching. We expected self-related benefit to increase communal sharing, and communal sharing to increase gratitude, which should result in an indirect effect of benefit on gratitude. Furthermore, we predicted that the same would not be true for equality matching.

### Method

#### Participants

Students from a Portuguese university in Lisbon (ISCTE-IUL) took part in this experiment, either in exchange for a €5 voucher or for course credit. Analyzes are based on data from 137 participants (62% of females, *M* ^age^  = 19.84, *SD* = 2.99). Ten participants were initially excluded because they did not believe in the cover story. All procedures were conducted according to the ethical guidelines and approved by the ethics board of the Scientific Commission of the hosting institution, Centro de Investigação e Intervenção Social (Cis-IUL).

#### Design

Benefit was manipulated between participants as either *self-related* (participant is the one who benefits) or *other-related* (someone else benefits).

#### Procedure

Participants arrived at the lab to participate in an experiment ostensibly about team work. They read and signed the informed consent and sat down at a computer while the experimenter opened an instant messaging (IM) window. Participants were told they were about to participate in a study related to team work with three other participants, who were in different rooms/cubicles, connected to a common chat room. Participants were assigned a nickname in the common chat room. All participants were given the nickname: “participant 1.” Participants were told that instructions would be given via IM.

The experimenter left the student alone in the cubicle, and started to give the instructions and take the role of participants 2 to 4:

[Experimenter]:

“We are a research group in Organizational Psychology. We are interested in the virtualization process for work teams, its advantages and disadvantages. Thus, we ask you to participate in the following task.

In this session we have four participants in different lab rooms, who will work in two teams.

Team one: Participant 1 (task duration: 30 minutes [15 minutes]) and Participant 2 (task duration: 15 minutes). Team two: Participant 3 (task duration: 15 minutes) and Participant 4 (task duration: 30 minutes).”

[Participant 2]:

“I would like to swap with participant 1 [*participant 4*] because I have free time in the next hour/*I like to participate in experiments*” (see Text S2 for more details in the design).

The experimenter agrees with the request. Participants are told that even though they are a team, the tasks have to be performed individually.

A new individual IM window is opened, and the participant receives the link to his/her task, together with the additional information that s/he should “leave the chat room and stay offline”. The other participants are also signed off.

When the participant opens the link, s/he is given the following instructions for the distracter task (line bisection task):

“On the next few screens you will be presented with several lines. The goal of this task is to find and to signal the midpoint of each of the horizontal lines. To successfully complete this task, both members of the team must signal correctly the midpoint of the line.”

After each trial, participants are given bogus feedback about the task. On eleven of the fourteen trials, a positive message is shown on the screen congratulating the participant because both team members have been successful. On three trials, a negative message states that someone has failed, along with some encouragement. Next, participants fill out the dependent measures, demographics, and at the end participants are thanked, debriefed and tested for suspicion.

#### Dependent Variables

Participants rated to what extent they felt *gratitude* towards the other member of the team on a 7-point Likert scale (1 =  not at all; 7 =  extremely). Moreover, we measured the *relational models*, specifically the *communal sharing* (α = .87) and the *equality matching* (α = .84) scales as in Studies 1 and 2, but regarding an imagined future relationship. All the items were presented in the future unreal conditional (e.g., “*‘What's mine is yours’ would be true of this relationship”*).

### Results

To test our main hypothesis, we performed a regression analysis with communal sharing and equality matching as predictors and gratitude as the dependent variable. As predicted, only communal sharing predicted gratitude (β = .33, *p*<.01) when controlling for equality matching (β<.1, *p* = *ns*).

Testing our second prediction with an ANOVA, we found a main effect of benefit on communal sharing, *F*(1, 135)  = 19.00, *p*<.001. Participants indicated more communal sharing in the self-related benefit condition (*M* = 3.54, *SD* = 1.26) than in the other-related benefit condition (*M* = 2.65, *SD* = 1.14).

Therefore, we tested the indirect effect of benefit on gratitude via communal sharing with a mediational analysis, as suggested by Preacher and Hayes [32]. The results revealed that communal sharing mediates the relation between benefit and gratitude (indirect effect of 0.49; 95% Confidence Interval [0.25; 0.87]; *p*<.001). Participants in the self-related benefit condition perceived the relationship with the unknown interaction partner as more communal sharing, which in turn increased gratitude. We tested the reverse path: benefit as the independent variable, gratitude as the mediator and communal sharing as the dependent variable. This path was not statistically significant (indirect effect  = 0.04; 95% CI [−0.09; 0.21]; *p* = *ns*). Additionally, we conducted the same analysis with equality matching as the mediator, however, the indirect path was not statistically significant (0.11, 95% CI [−0.02; 0.34]).

### Discussion

We again replicated the finding that applying the communal sharing model increases gratitude. Furthermore, we find that receiving a benefit directly increases the likelihood of imagining a future relationship with an unknown fellow student as communal sharing. Moreover, responding to a non-face-to-face interaction with the idea of relating in a communal way increased gratitude felt towards the interaction partner. This pattern did not extend to equality matching. Based on this finding, we propose that non-contingent benefits signal a communal way of sharing resources to which individuals respond by applying a communal sharing model. Subsequently, they feel grateful for the benefit to the extent that they apply the communal sharing model.

## General Discussion

Across three studies, we found that gratitude for a benefit received was predicted by perceived communal sharing with the benefactor. This was the case when we asked participants to imagine receiving a benefit from a new acquaintance (Study 1), when we asked them to recall a large benefit received from a friend (Study 2) and when they received a benefit from a stranger in an experimental situation. In all of these cases, scores on a scale measuring the amount of communal sharing in the relationship predicted gratitude also when controlling for other relational models, but no other relational model predicted gratitude when controlling for communal sharing. Specifically, in all three studies, we also measured equality matching, and in Studies 1 and 2 we measured authority ranking in addition.

Taken together, these results show that communal sharing is an important link connecting benefits and gratitude: people are grateful for benefits that are offered within a communal relationship. The communal relationship can either be already established before the benefit (see Studies 1 and 2) or the benefit can signal communal intentions of the benefactor and thereby activate the communal sharing model in the receiver (see Study 3).

### Relational Models Theory

We based our hypothesis on the observation that only in communal sharing, benefits are not provided out of a direct relational obligation or entail such an obligation. However, whereas equality matching clearly entails relational obligations, the case is not as obvious for authority ranking. Within authority ranking relationships, superiors take responsibility for subordinates, for example by helping, protecting or teaching them. Thus, ideally, superiors are responsive to the needs of subordinates. For example, when the relationship with a deity is perceived as one where the deity has all resources and no relational obligations, any benefit the subordinate human receives should lead to gratitude. However, in Western cultures, social norms have shifted in the last decades such that authority ranking is considered as illegitimate in many contexts where it used to be the main model a century ago. In our results, we also found less authority ranking (3.97) than equality matching (4.31) or communal sharing (4.80) across Studies 1 and 2 [33]. Thus, to test whether authority ranking can lead to gratitude, future studies should use contexts or groups where authority ranking is perceived as a legitimate model by participants.

Relational Models Theory assumes that the function of social emotions is to motivate optimal relational equilibria [8]. Gratitude can function to strengthen bonds between partners, whereas anger can motivate a relationship's termination. RMT also predicts that these social emotions are rooted in “relationship-specific heuristics” [8]. This means that social emotions reflect a relational state, and they function to promote behavior matching the specific relational model [8]. Our findings are in line with this theoretical claim. We found that within communal sharing, gratitude reflects the state of having received a benefit [2], [3]. This presumably indicates that the relationship works well, and is worth keeping up.

There is a fourth relational model, market pricing. Market pricing is based on proportionality, and it thereby allows the exchange of different kinds of resources, such as money and goods, crime severity and prison time, or apples and pears. Whenever exchange ratios can be specified (2 apples for 1 pear), market pricing is the underlying model. In the present set of studies, we did not include a measure of market pricing. We knew from prior studies that it was not common for relations among friends, fellow students and family in our student population [34]. Furthermore, it should be the least likely to evoke gratitude, as it is usually based on the most explicit, formal kinds of agreements where obligations are strong, and can even be enforced by law. However, future research should include market pricing as well.

In the introduction, we argued that communal sharing leads to gratitude because the communal partner is perceived as intending to fulfill the partner's needs. However, in the present studies, we did not measure perceived intentions. Rather, we measured the perception of the relationship. This is because, according to relational models theory, the representation of a relationship is not primarily about the other person's intentions, nor about one's own intentions, but about the way people respond to each other. Take, for instance, the situation where a person tries to fulfill your needs, but you do not want to be communal with her. In that situation, you would also perceive the intention as a communal intention, but you would not apply a communal sharing model, so you should not feel grateful. Accordingly, the communal sharing model should be a better predictor of gratitude than perceived intentions. Nevertheless, future studies should include a measure of perceived intentions, and investigate the process through which communal sharing increases gratitude.

In what follows, we will discuss further implications of our findings with reference to the additional assumptions that we tested. However, each of these additional assumptions was only tested in one of the studies, so our conclusions are somewhat more preliminary than for the main finding. Future research is needed to replicate these additional findings.

### The Communal Sharing Model

Communal sharing is defined as a mental representation of a social relation [7]. The construct encompasses communal norms, mutual intentions to fulfill each other's needs, and the expression of the relation, for example, by sharing food, touching, or being close to each other. The communal sharing scale that we used assesses the perceived extent of communality in the relationship. It incorporates all these aspects (Appendix S1) and the good reliability as well as research findings that these items load on one factor, support the theoretical construct [10]. Communal norms, such as a sense of unity, concern for each other's welfare, treating all members of the relationship as equivalent, and free sharing of resources, are also part of many other, prior constructs. For example, a shared concern for each other's welfare and not keeping track of what is given and received are also defining characteristics of a communal orientation [27], [35]. Similarly, communal strength is described as a construct highly dependent on the adherence to communal norms. The cost that one is willing to incur to benefit a communal partner will be higher in relationships where more communal norms are applied [23], [36].

A broad measure of communal sharing should therefore predict gratitude, which depends on the perceived intentions of the relational partner, and whether they are valued. It should also predict one's own motivation to meet the needs of the relationship partner, i.e. communal strength. In other words, the relation between gratitude and communal strength should be fully explained by communal sharing. Our results indeed indicated that this was the case, and that communal sharing independently predicted communal strength and gratitude. Therefore, we suggest that a mental representation of communal sharing relationships precedes both communal strength and gratitude in a relational context. However, we obtained these results in a cross-sectional study. It would be interesting to see if communal strength is also longitudinally predicted by communal sharing, just as gratitude is.

When one focuses on the functions of gratitude rather than on its predictors, prior findings show that gratitude and the expression of gratitude serve to drive engagement in communal relations. Being grateful, expressing and receiving gratitude, all increase motivation to behave according to communal norms [6], [30], [37]. These findings can be interpreted in a relational models framework: Equality matching and market pricing relations can be sustained with behavioral intentions. For example, if other parents have taken your child or you want them to take your child some evening you might form an intention to invite their child over to fulfill or create a relational obligation, respectively. Thus, the behavioral intention drives engagement in the relationship and reminds one of one's obligations. In communal sharing, however, the benefits given and received do not correspond. Therefore, the feeling of gratitude can take the role of a reminder to be kind and generous to the relational partner. It is therefore conceivable that gratitude can also enhance communal sharing and communal strength, however, more research is needed to find out when and how this happens. Prior research shows that the expression of gratitude is important for this influence [37]. This might point to the importance of factors such as awareness, self-perception, or sharing of the gratitude. Furthermore, frequent gratefulness might enhance communal sharing and communal strength.

### Theories of Gratitude

In Study 2, we found that communal sharing also predicted the gratitude felt regarding the relational partner in general, not just regarding particular benefits. At Times 2 and 3, participants were reminded of a relational partner and indicated their gratitude regarding this person. Gratitude at Time 3 was predicted by communal sharing reported at Time 2.

These results fit into two different theoretical approaches of gratitude: Gratitude as a reaction to responsive benefits, i.e. benefits which respond to one's needs [5], [6], and gratitude as a reaction to important relationship partners [19], [37], [38]. Our main result was that gratitude for benefits depends on communal sharing. This is in line with the responsiveness account of gratitude, if one assumes that the perception of the responsiveness of a benefit is based on communal sharing. On the other hand, individuals are suggested to feel generalized gratitude regarding relational partners [19]. We also found this pattern in Study 2, where, without evoking a benefit, gratitude was predicted over time by communal sharing. Therefore, our results seem to suggest that both approaches can be integrated: communal sharing predicts gratitude because it means that the communal partner is motivated to meet one's needs both for a current benefit and also across time, in the past, present, and future.

However, in Study 2, we only measured relational models and gratitude regarding the relational partner after participants had already recalled a concrete benefit from that partner. We believe that the three weeks between the measurements minimized the influence of that recall. Nevertheless, a limitation of this study is that we cannot exclude that somehow the memory of the recalled benefit influenced our measures at Times 2 and 3.

### Non-Contingent Benefits as Cues to Communal Sharing

Finally, our results show that certain benefits can also increase the communal sharing perceived in a relationship, thereby having an indirect effect on gratitude via communal sharing. In Study 3, receiving a benefit did not increase gratitude compared to not receiving a benefit. Only those who perceived more potential for communal sharing with the benefactor as a result of receiving the benefit felt more grateful towards her.

We believe that a non-contingent benefit functions as a cue, easy to interpret as showing a communal intention. Based on previous communal experiences, this should prompt an application of the communal sharing model [9].

In the absence of any additional information about the benefactor, the likelihood of applying a communal sharing model will probably be influenced by individual differences in the tendency to be communal with others. In line with this, the self-reported importance of having communal sharing relations correlates moderately (.34) with the average communal sharing score for a sample of relations [39]. However, the fact that communal sharing in Study 3 was influenced by the benefit manipulation shows that situational cues also determine which model is applied, not only inter-individual differences. Furthermore, there is ample evidence that with the same relational partner, different models can be applied in different contexts, even when a dominant model can be identified [7].

### Conclusion and Outlook

Positive social emotions like gratitude have been found to be very important for motivating individuals to be empathic, caring, and considerate, thereby contributing to the cohesiveness and functioning of couples, families, and larger social units [40]. It is therefore important to learn more about these emotions, and the current research builds on and integrates recent work towards this goal by proposing a new predictor of gratitude, the relational model of communal sharing. Convergent evidence that communal sharing predicts gratitude was found in a cross-sectional, longitudinal and an experimental design. These findings are encouraging for testing hypotheses about relational models and other social emotions. According to Fiske [8], emotions play specific roles in motivating the constitution, maintenance, retribution and termination of relationships depending on the perceived relational model. It is therefore worthwhile for future research to study the role of relational models in emotions like awe, admiration, pride, anger or shame. Furthermore, relational models can be evoked by different types of cues, some of them embodied, like touch or commensalism as cues for communal sharing. Relational models theory thus allows novel predictions for a causal path from nonverbal behavior to social emotions via relational models. Studying these will help understand better the pervasive effects of nonverbal cues and the automatic nature of relationship regulation.

## Supporting Information

Appendix S1 Relational Models Scales (communal sharing, authority ranking and equality matching subscales) used in Studies 1–3.
(DOCX)

Text S1 Additional Analyses for Study 1.
(DOCX)

Text S2 Additional information on design for Study 3.
(DOCX)
