# Supplementary material for: Gratitude Depends on the Relational Model of Communal Sharing
Source: PLoS One. 2014 Jan 22;9(1):e86158. doi: 10.1371/journal.pone.0086158 (PMC3899114; doi:10.1371/journal.pone.0086158)
Supplement: Appendix S1 — Relational Models Scales (communal sharing, authority ranking and equality matching subscales) used in Studies 1–3. (DOCX) [file pone.0086158.s001.docx]

Appendix S1: Relational Models Scale (communal sharing, authority ranking and equality matching subscales) used in Studies 1-3.

**Communal sharing**: This person would “give the shirt off their back” for you; “What’s mine is yours” is true of this relationship; You share food with this person; If this person needed help, you could cancel plans to give it; “One for all and all for one” is true of this relationship; What happens to this person is almost as important to you as what happens to you.

**Authority ranking**: One of you calls the shots in this relationship; One of you takes most of the initiatives; One of you backs the other up in this relationship; One of you takes most responsibility in this relationship; One of you protects the other; One of you tends to lead.

**Equality matching**: Your relationship is organized on a 50 *: 50* basis; You are pretty equal in the things you do for each other; If one of you does something for the other, the other tries to do the same thing in return next time; You often take turns doing things; If you share something, you divide it down the middle; You more or less keep track of favors and obligations.
