# Supplementary material for: Gratitude Depends on the Relational Model of Communal Sharing
Source: PLoS One. 2014 Jan 22;9(1):e86158. doi: 10.1371/journal.pone.0086158 (PMC3899114; doi:10.1371/journal.pone.0086158)
Supplement: Text S1 — Additional Analyses for Study 1. (DOCX) [file pone.0086158.s002.docx]

Text S1: Additional Analyses for Study 1.

To test whether our two samples differed with regard to the hypothesized relationship, we repeated this regression analysis with sample as a factor. We added the main effects of the three relational models, the main effect of sample, and the three computed interactions (each relational model with sample). As expected, the only predictor of gratitude was the main effect of communal sharing (β = .26, *p* = .009). Furthermore, the interaction between authority ranking and sample showed a marginal effect (β = -.16, *p* = .06), which means that for the Portuguese sample, authority ranking also predicted gratitude (β = .26, *p* < .05). Given that this interaction was unexpected and not significant, we needed to see whether it would replicate in a second study.

For all multiple regressions reported in Study 1, we confirmed with the Variance Inflation Factor that our predictors showed low multicollinearity.
