# Supplementary material for: Gratitude Depends on the Relational Model of Communal Sharing
Source: PLoS One. 2014 Jan 22;9(1):e86158. doi: 10.1371/journal.pone.0086158 (PMC3899114; doi:10.1371/journal.pone.0086158)
Supplement: Text S2 — Additional information on design for Study 3. (DOCX) [file pone.0086158.s003.docx]

Text S2: Additional information on design of Study 3.

We set the times for participant 1 such that he/she would always have 15 minutes in the end.

The reasons for benefit were counterbalanced across participants and were varied for exploratory purposes. Because we did not expect and did not find any differences between the two reasons, we collapsed across this factor in all analyses.
